# Morbid Obesity in Disasters: Bringing the “Conspicuously Invisible” into Focus

**DOI:** 10.3390/ijerph13101029

**Published:** 2016-10-20

**Authors:** Lesley Gray, Carol MacDonald

**Affiliations:** 1 Department of Primary Health Care & General Practice, University of Otago, Wellington 6242, New Zealand; 2 Joint Centre for Disaster Research, Massey University & GNS Science, Wellington 6021, New Zealand; carol.macdonald@xtra.co.nz

**Keywords:** obesity, vulnerability, disaster risk reduction, natural disasters, emergency planning, preparedness

## Abstract

It is a frightening reality for some people to be caught up in the midst of a disaster, alone and vulnerable due to their relative size, shape or weight. A literature search failed to find any empirical reports of data specific to body mass index (BMI) in disaster situations. A handful of largely anecdotal reports described situations in which people categorised as morbidly obese were negatively impacted in disasters because of their size and/or weight. While a small number of toolkits and training resources were found, there remains a paucity of research in relation to obesity and emergency planning or disaster risk reduction. This is somewhat surprising, considering the concern about increasing levels of obesity globally. Research is urgently needed to prioritise and address the specific considerations of people with morbid obesity and how communities plan, prepare, respond, and recover from disasters and public health emergencies.

## 1. Introduction

Imagine you are caught up in the midst of a disaster, alone and vulnerable, and the only difference between you being rescued or left behind is your relative size, shape or weight. This has been the frightening reality for some people, yet there is little published literature concerning obesity in the context of disasters.

A literature review failed to find any empirical reports of data specific to body mass index (BMI) casualties or fatalities in disaster situations. There are, however, a handful of largely anecdotal descriptions of situations in which people categorised as morbidly obese were negatively impacted in disasters because of their size and weight. 

## 2. Disturbing Accounts

Perhaps the earliest account relating to natural disasters and obesity dates from AD 79 [1] (p. 127). Pliny the Elder, lived within sight of Vesuvius across the Bay of Naples. When Vesuvius erupted, he took a small ship across the Bay for a better view. On departure, Pliny learnt of a friend trapped near the eruption, however with adverse winds and waves he was forced to shelter and wait with friends who had joined the “rescue”. Falling ash and stone threatened to bury them and Pliny had trouble breathing, was overcome by gas, ash and exertion and died. The account concluded that Pliny’s death, while his friends survived was likely due his corpulence, overexertion and weak constitution.

In more recent times, a number of documented cases involve people with obesity in the aftermath of Hurricane Katrina in 2005. This hurricane caused severe destruction along the Gulf coast from central Florida to Texas, devastating New Orleans when flood protection measures were breached. At Memorial Medical Center (MMC), 34 patient deaths resulted in three staff being charged with second-degree murder in relation to four deaths. Reports indicate that one of those patients, awaiting surgery for a non-life-threatening condition, was obese and paralyzed. It is recorded that he appealed to his nurse several times during the evacuation “don’t let them leave me behind” [2] (p. 297). The care group discussing patient evacuations concluded that this particular patient was approximately 170 kg and too heavy to be evacuated. He was apparently alert and conscious when he was administered a lethal dose of morphine and midazolam, drugs he was not receiving for routine care. Other medical staff who led evacuations later claimed that they would have found a way to evacuate this patient had they been made aware of his presence.

Another MMC patient, reportedly close to death and being treated for comfort only, was visually assessed as around 159 kg. The task of moving her down the stairs was considered impossible. Two days after Katrina hit, an exhausted specialist is quoted as saying “I gave her medicine so I could get rid of her faster, get the nurses off the floor,” as the nurses were needed elsewhere [3]. 

When Katrina struck, one other patient in the MMC intensive care was recovering from heart problems and multiple operations. Described as obese, he lay motionless on a stretcher, covered in sweat and almost nothing else. He was the last living patient to leave the hospital, left until all other patients had been evacuated from fear of blocking the evacuation route [3].

An account from a New Orleans hospital involved 12 staff members taking almost two hours to carry one patient with obesity down an emergency stairwell [4]. A similar situation occurred at Tulane University Hospital involving two patients weighing over 180 kg, along with two patients requiring 180 kg of equipment each. The Tulane patients were carried down six to eight flights of stairs in darkened stairwells in what was described as a “Herculean effort by their caregivers” [5].

In 2012, Superstorm Sandy wreaked havoc on the East Coast of America. In the 48 hours following Superstorm Sandy, all but two patients were evacuated from Bellevue Hospital Center (BHC) in New York City. One of these patients had a BMI of 81.4 kg/m^2^, weighing 263 kg. It was documented that she was too wide for the evacuation sled and this, along with safety concerns for the patient and evacuation personnel, prohibited evacuation down the 15 flights of stairs. Ramme, Shaleen and McLaurin [6] specifically focused on the significance of this patient’s relative size, shape and weight as the defining factors for her being left behind. However, in another account of the BHC evacuation, this patient’s size and weight were not mentioned. Rather her medical condition was cited as the primary reason for the delay in evacuation until elevators were available [7]. In fact, she was hospitalised for a knee injury and had been deemed medically stable for movement if this had been possible [6]. 

## 3. Unique Challenges

These cases highlight the significant challenges that patients with morbid obesity present in a disaster situation. Under normal circumstances managing patients with morbid obesity requires increased resources such as personnel, supplies, and specialist equipment [8,9]. They present unique challenges in emergency management, including rescue and evacuation, adequate rescue transportation, challenges in critical care, and suitable equipment, including beds and chairs in shelter settings [10,11].

In a disaster, various co-morbid conditions such as hypertension, deep vein thrombosis, diabetes, respiratory difficulty, and hypoxemia, may increase their likelihood of suffering harm [8]. People with poor health, disabilities, and chronic disease may be referred to as vulnerable groups, or populations of concern [12] and are at increased risk of adverse health outcomes resulting from natural disasters [13]. People receiving care in the home have also been identified as vulnerable during disasters due to high rates of chronic disease, cognitive impairment, functional limitations, and physical disabilities as well as dependence on life-saving treatments and equipment [14]. Obesity has been included as a chronic condition [12] and can be affected by and potentially increase a person’s risk for certain diseases and disabilities [15,16].

Disabled people have been identified as disproportionately vulnerable to natural hazards in relation to the consequences of social disadvantage, poverty and structural exclusion [17]. Whilst there has been increased focus on such populations in relation to access and functional needs regarding disaster preparedness over the last decade [18] these populations have also been found to be less likely to have household preparedness items [19], and to be less prepared than populations without disabilities [13]. Conversely those populations with medical conditions are more likely to have medication supplies than less vulnerable counterparts [19], and people who use specialised equipment such as wheelchairs are likely to be more prepared than people who do not use such equipment [13]. 

Morbid obesity in itself can be completely disabling and can inhibit a person’s ability to help with their own movement in an emergency [20], may require a high degree of assistance, and people with morbid obesity may be more at risk of hospitalization during and following a disaster event [21]. In the often resource-constrained setting of disasters, normal support and care for such patients can be jeopardized [8].

There is some literature detailing exacerbation of risk for persons with obesity in certain public health emergencies such as the 2009 influenza A(H1N1) pandemic [22,23,24,25,26,27], with disproportionate hospitalisation and mortality for people categorised as obese (BMI ≥ 30 kg/m^2^) or morbidly obese. The issue of obesity as a consequence of the effects of disasters, particularly involving prenatal maternal stress and early childhood development has also been explored [28,29].

A high prevalence of morbid obesity (approximately 20%) among those affected by Hurricane Ike in 2008 reportedly strained the evacuation and emergency shelter systems [8]. This issue has led some U.S. relief organizations to require special-needs shelters to accommodate people with morbid obesity (BMI ≥ 40 kg/m^2^), thereby creating a strain on those resources as well [30].

Most of the small amount of literature found relates to disaster response and not the implications of obesity for disaster risk reduction (DRR). A small number of toolkits and training resources were found in the grey literature [21,31,32,33,34], including a 2-page factsheet highlighting a number of planning considerations for those with extreme obesity in disasters and public health emergencies [35]. There is little evidence that such considerations have been translated into emergency management planning or practice. A 2012 survey of Pennsylvania hospitals found that more than one-third (36.5%, *n* = 23 of 63) of the respondents did not have an evacuation plan in place for moving patients with morbid obesity to a safe location in the event of an emergency [36].

One study focused on disaster as a means to work with communities to promote the reduction and management of weight in order to be better able to respond to natural disasters in the future [37]. This study considered individual determinants and subsequent household response following the 8.1 magnitude earthquake and subsequent tsunami in American Samoa in 2009. The study found that while people were very adaptive and able to evacuate quickly to safe areas following the earthquake and before the tsunami reached shore, some residents were hindered in their ability to evacuate due to obesity related health issues limiting mobility of individuals or assisting family members [37] (p. 367). The prevalence of obesity in the American Samoa was reported as high as 74.6% in a 2004 survey [38].

The USA, Mexico and New Zealand have the highest levels of measured obesity in the Organisation for Economic Co-operation and Development (OECD) [39], at 35.3%, 32.4% and 31.2% respectively. These countries also have a high number of potential natural hazards and experience of natural disasters. The paucity of research considering obesity in relation to emergency planning or DRR is therefore somewhat surprising, considering the concern about increasing levels of obesity globally. 

It is less surprising that obesity does not feature as a disaster-related issue in countries with relatively low levels of obesity, such as Japan (3.6% measured data) [39], or Italy (10.4%, self-reported data) [39] despite the occurrence of significant disaster events.

New Zealand sits over the so called “ring of fire” [40], with frequent seismic activity. Obesity amongst adults in New Zealand is increasing, with indigenous Māori and Pacific adults experiencing disproportionate levels of morbid obesity (10% and 20% respectively) [41]. 

At the time of writing (September 2016) a magnitude 7.1 earthquake was experienced off the East Cape of New Zealand’s North Island, an area with the highest proportion of Māori population. A swarm of earthquakes and a tsunami warning followed. Had a tsunami on the scale of that experienced by Japan in 2011 eventuated, what would that have meant for those of the local population directly or indirectly affected by obesity? How many people may have been inhibited in their efforts to move quickly to higher ground as a direct consequence of their size or weight, or of family members or those being cared for?

## 4. Conclusions

This commentary raises the question of the vulnerability of people with morbid obesity and the implications this has for emergency planning and DRR generally. People with morbid obesity are a vulnerable population and planning is needed to mitigate the effects that disasters have on them. Prior planning can address appropriate health care and psychosocial support needs for people with morbid obesity but also promote self-care and resilience and reduce the need for crisis intervention during and following an event. 

In terms of obesity policy, countries with high levels of obesity, including the USA, UK and New Zealand have been called to action and pledged commitment to tackle obesity rates over the last two to three decades. Despite this, globally there are no significant accounts of obesity reduction over the last 33 years [42]. Most of the action has focused on individual, community, clinical and educational interventions and have failed to address environmental factors [43,44].

Until the tide of this “obesity tsunami” is turned we can expect more people in our communities to experience increased vulnerability during disasters as a result of morbid obesity, associated comorbidities and disability. Strategies for our largest members of the population are required and are notably absent from current frameworks intended to support DRR efforts. 

A research participant in a morbid obesity simulation study currently being undertaken in New Zealand [45] observed when wearing a suit to simulate morbid obesity:

“It’s weird that you’re invisible, and yet you’re so conspicuous.”

(Participant 003, female)

If we are to avoid situations such as those described earlier in this commentary, those who are currently “invisible” must become a focus of our attention. Research is urgently needed to prioritise and address the specific considerations of people with morbid obesity in how we plan, prepare, respond, and recover from disasters and public health emergencies. 

### Limitations

As a scoping review, this paper did not appraise the quality of evidence in primary research reports and was limited to reports and documents published in English. We may have omitted, for example, potentially important documents relating to Mexico’s emergency preparedness information, written in Spanish. 
